# Pancreatic juice outflow in pancreatojejunostomy monitoring with the inter-anastomosis drainage tube; a retrospective observational study

**DOI:** 10.1186/s12893-022-01669-x

**Published:** 2022-07-14

**Authors:** Osamu Shimomura, Tatsuya Oda, Yoshihiro Miyazaki, Kinji Furuya, Manami Doi, Kazuhiro Takahashi, Jaejeong Kim, Shoko Moue, Yohei Owada, Koichi Ogawa, Yusuke Ohara, Yoshimasa Akashi, Tsuyoshi Enomoto, Shinji Hashimoto

**Affiliations:** 1 Department of Surgery, Clinical Sciences, Faculty of Medicine, University of Tsukuba, Ibaraki, Japan; 2 Department of Gastrointestinal and Hepato-Biliary-Pancreatic Surgery, Faculty of Medicine, University of Tsukuba, Ibaraki, 305-8575 Japan

**Keywords:** Blumgart, Drainage, Pancreatic Fistula, Pancreatoduodenectomy, Pancreatojejunostomy

## Abstract

**Background:**

Pancreatic fistula remains the biggest problem in pancreatic surgery. We have previously reported a new pancreatojejunostomy method using an inter-anastomosis drainage (IAD) suction tube with Blumgart anastomosis for drainage of the pancreatic juice leaking from the branched pancreatic ducts. This study aimed to evaluate the postoperative outcomes of our novel method, in pancreatojejunostomy and investigate the nature of the inter-anastomosis space between jejunal wall and pancreas parenchyma.

**Methods:**

This retrospectively study consist of 282 pancreatoduodenectomy cases, including 86 reconstructions via the Blumgart method plus IAD (B + IAD group) and 196 cases reconstructed using the Blumgart method alone (B group). Postoperative outcomes and the amylase value and the volume of the drainage fluids were compared between the two groups. The IAD tube was placed to collect amylase-rich fluid from the inter-anastomosis space during operative procedure between the jejunal wall and pancreatic stump.

**Results:**

The daily IAD drainage volume and the amylase level was significantly higher in patients with a soft pancreas (vs hard pancreas; 16.5 vs. 10.0 mL/day, *p* = 0.012; 90,900 vs. 1634 IU/L, *p* < 0.001, respectively). The mean amylase value of IAD collection in 86 cases of B + IAD group was 63,100 IU/L. The incidence of clinically relevant pancreatic fistula grade B and C (23.2% vs. 23.0%, *p* = 0.55) and the hospital stay was similar between the groups (median 17 vs. 18 days, *p* = 0.55). In 176 patients with soft pancreas, the incidence of pancreatic fistula grade B and C (33.3% vs. 35.3%, *p* = 0.67) and the hospital stay was also similar between the groups (median 22.5 vs. 21 days, *p* = 0.81).

**Conclusions:**

Positive effect of the IAD method observed in the pilot cases was not reproduced in the current study. IAD tube objectively demonstrated the existence of amylase-rich discharge at the anastomosis site, and countermeasures to eliminate this liquid are highly desired for preventing pancreatic fistula, especially in patients with soft pancreatic texture.

*Trial registration* Retrospectively registered

**Supplementary Information:**

The online version contains supplementary material available at 10.1186/s12893-022-01669-x.

## Background

Pancreatic fistula (PF) is still a major issue after pancreaticoduodenectomy (PD) and it is associated with a high mortality rate even with recent advances in surgical procedures and postoperative management [1–3]. The rate of clinically relevant postoperative PF (POPF) was higher than 10% in previous studies [2, 4], and it is an unsolved problem in the field of pancreatic surgery.

Recent improvements in the surgical technique—Blumgart anastomosis—proposed by Blumgart et al., enables a tight adaptation between the jejunum wall and fragile pancreatic parenchyma, protecting the thin pancreatic coat with jejunal serosa [5]. Several modifications in suture methods have been reported [6–8], and this anastomosis is now widely accepted. However, Blumgart anastomosis did not significantly reduce clinically relevant POPF compared with conventional interrupted sutures based on a randomized controlled study reported by Hirono et al. [9].

A soft pancreatic consistency with the normal ability to produce pancreatic juice is a well-known risk factor for POPF after PD [4, 10]. For a soft pancreatic texture, countermeasures are required to prevent pancreatic juice efflux from both the main pancreatic duct (MPD) and branch duct. The main causes of PF are divided into two categories. The first is leakage from the anastomosis of the MPD and mucosal membrane, and the second is leakage from small openings of branch pancreatic ducts in the parenchymal stump. Although Blumgart anastomosis enables tight adhesion between the jejunal wall and pancreatic stump, it may not be enough to reduce leakage from the branch duct of the pancreatic parenchyma. Undesired outflow of pancreatic juice initially collects in the inter-anastomosis space between the pancreatic stump and jejunum serosal wall. Subsequently, the juice flows out from the inter-anastomosis space into the peritoneal cavity, leading to a long-standing PF. Invagination and dunking-type reconstruction, the conventional approaches for preventing leakage from the pancreatic stump have been developed, in which the pancreatic stump invaginates into one large jejunal or gastric hole. The drawback of these methods is that activated digestive juice caused by a mixture of pancreatic juice, bile juice, and intestinal flora could induce severe PF.

Once a PF has developed, the healing of the PF requires the successful closure of the MPD and jejunal mucosa anastomosis and/or stopping the outflow of pancreatic juice from the branch duct. Evidence suggests that creating a spontaneous drainage route to the internal jejunal lumen is a key factor in the healing process [11, 12]. These findings indicate that the creation of a drainage route between the inter-anastomosis space and the jejunal lumen at the time of reconstruction might collect the effused juice and prevent PF.

Here, we present the postoperative outcomes of our strategy using a novel add-on method for pancreatojejunostomy. Our method involves the placement of a single-suction drainage tube into the space between the pancreatic stump and jejunum wall, namely the inter-anastomosis drainage (IAD) tube. No methods have been developed to provide information about the leakage of pancreatic juice from the stump. Our method highlights the importance of an undiscovered inter-anastomosis space in pancreatojejunostomy.

## Methods

### Patient background and data collection

This is a retrospective study including 282 patients who underwent PD with Child-type reconstruction using modified Blumgart-type pancreatojejunostomy [7] at a single institution in Japan (Department of Gastrointestinal and Hepato-biliary-pancreatic Surgery, University of Tsukuba Hospital) from July 2010 to June 2020. Among the 282 patients, 86 underwent Blumgart with IAD (B + IAD group), and 196 patients only underwent the Blumgart method (B group). B + IAD was performed between January 2014 and February 2018, and the Blumgart-only (B group) procedure was performed before and after this period. Clinical data and pre- and postoperative data were reviewed from the medical records and analyzed retrospectively. A subgroup analysis was performed for patients with a soft pancreatic texture. All patient data sets were collected after approval by the institutional review board of Tsukuba University Hospital (R01-030).

### Outcome assessment

POPF was diagnosed according to the classification of the International Study Group on Pancreatic Fistula (ISGPF) [13]. Clinically relevant POPF was defined as grade B or C. Grade B required a change in the postoperative management; drains were either left in place for more than 3 weeks or replaced by endoscopic or percutaneous procedures. Grade C required reoperation or led to single or multiple organ failure and/or mortality attributable to PF [13]. The status of the pancreatic parenchyma (soft or hard pancreas) was determined by subjective palpation of the operator. The size of the MPD was measured at the presumed surgical transection line on preoperative contrast-enhanced computed tomography.

### Operative procedure

#### Pancreatic transections

Pancreatic transections were performed using a sharp scalpel, and any notable bleeding and recognizable openings of small branches of the pancreatic ducts were ligated with 5-0 monofilament stitches. Hemostasis of small hemorrhages was achieved using electrocautery.

#### Placement of the inter-anastomosis drainage tube

**Fig. 1:**
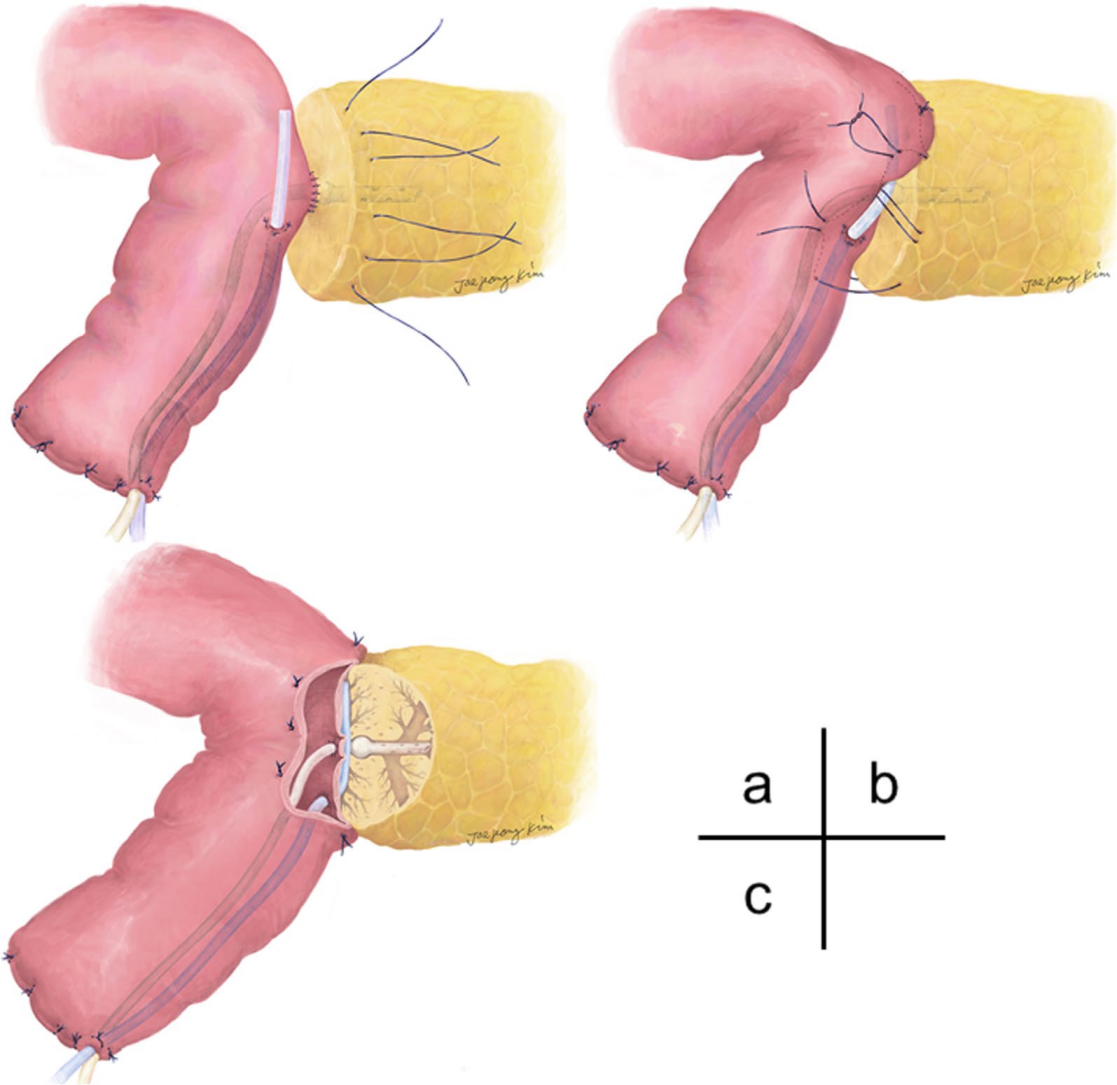
The placement of the inter-anastomosis drainage (IAD) tube. After completing the duct-to-mucosa anastomosis, the IAD tube was placed at the interspace between the jejunal wall and pancreatic parenchyma through the end of the jejunum (**a**). A 10 Fr silicone tube (BLAKE Silicone Drains-Hubless, Ethicon) was arranged in an “I” shape and was inserted into the inter-anastomosis space. The external drainage tube of the MPD stent was placed and fixed, and Blumgart mattress sutures (3-0 PDS-II, Ethicon) were ligated by wrapping the IAD tube inside (**b**). The IAD tube was completely enveloped by the jejunal wall and pancreatic parenchyma (**c**). Reinforcing 4-0 PDS-II sutures were occasionally added to both edges in order to ensure a water-tight seal. At the end of the surgery, the IAD suction tube was connected to a low-pressure continuous-suction device

After completion of the duct-to-mucosa anastomosis, the IAD tube (n = 86) was placed in the interspace between the jejunal wall and pancreatic parenchyma (Fig. 1a). This procedure was designed to extract undesirable pancreatic juice from the inter-anastomotic space using an active suction system. Briefly, an IAD tube (10 Fr. The BLAKE Silicone Drains-Hubless, Ethicon, code 2226, NJ, USA) was positioned such that the short-channel-suction part was located in the inter-anastomosis space between the pancreas and jejunum (Fig. 1b), the middle extension part was located in the inner lumen of the jejunum, and the distal end protruded from the end of the jejunal limb, which is commonly referred to as Witzel’s end. The channel part of the tube was originally in the shape of a “+” sign with 4 flaps, which were arranged into a thin and flat “I” shape by removing the anterior and posterior flaps, and the length was adjusted to the width of the pancreatic parenchyma (usually 2.5–3.5 cm). A water-tight seal between the active IAD tube and jejunal wall was used to block the reverse flow of the contaminated intestinal juice into the inter-anastomotic space. For this purpose, the extra-abdominal end of the tube was cut diagonally, passing the sharp end through a small jejunal hole to ensure a tight seal. After creating passive IAD holes or after the placement of the active IAD tube, three ties using the Blumgart sutures were made firmly at the anterior serosal wall of the jejunum, and the IAD tube was completely enveloped by the jejunal walls (Fig. 1c). Reinforcing 4-0 PDS-II sutures were occasionally added to both edges. At the end of the surgery, two prophylactic peritoneal drainage tubes were placed at the superior and inferior sites of the pancreatojejunostomy, and the active IAD tube was connected to a low-pressure, continuous suction device (100 mL Bulb Suction Reservoir, Ethicon, code 2160, NJ, USA). All procedures during PD were performed by or under the direction of three surgeons (O.S., S.H., and T.O.).

### Postoperative management

The fluid amylase levels from the peritoneal drainage tubes were monitored on postoperative day (POD) 1, 3, and 5. On POD 4–5, the tubes were removed for patients with ISGPF grade [14] None or BL (biochemical leakage) and were replaced with new 18–22-Fr silicon tubes (Fuji Systems Co., Tokyo, Japan) using interventional radiographic techniques for patients with possible ISGPF grade B or C. In the IAD group, the volumes drained by the IAD tube were measured daily, and the amylase levels were measured once on POD 1–3. After that, the IAD tube was connected to a suction bag and the collected fluid was carefully observed every day and removed after confirming that the PF had healed. The nasogastric tubes were removed on POD 1 and liquid oral intake began on POD 1, followed by the administration of a liquid diet beginning on POD 2–3 and solid food beginning on POD 4–7. If PF was present, the external drainage tube was evaluated and replaced using contrast examination. Further, no contrast materials were used for the IAD tube or pancreatic duct stent.

### Statistical analysis

Differences between the two groups (i.e., group B vs. group B + IAD) were statistically analyzed using SPSS version 26 (IBM, Armonk, NY, USA). Categorical variables were analyzed using the chi-squared test or Fisher’s exact test when 20% of the expected frequencies were less than or equal to 5. Continuous variables are presented as both the mean (SD) and median (range) and were analyzed using the Student’s *t*-test and Mann–Whitney *U* test for normally distributed and non-normally distributed variables, respectively. All *p*-values from two-tailed tests were considered statistically significant at *p* < 0.05.

## Results

### Demographics

A total of 282 patients underwent PD during the study period, including 86 patients in the B + IAD group and 196 patients in the B group. The demographics and summary of clinical outcomes are shown in Table 1. There were no significant differences in preoperative factors, such as age, sex, diagnosis, prealbumin value, diabetes, preoperative biliary stenting, and physical status (American Society of Anesthesiologists). The operation time was significantly longer in the B + IAD group than in the B group; however, there were no significant differences in blood loss and the rate of required portal vein reconstruction. Among the 282 patients, 176 had a soft pancreatic texture. A summary limited to the soft pancreatic texture is shown in Table 2; the patient characteristics did not change. The operating time was also significantly longer in the B + IAD group (507 vs. 458 min) for those with a soft pancreas.

**Table 1:** The summary of total 282 patients of Blumgart with IAD (B + IAD) and Blumgart only (B)

		B + IAD	B	*p* value
Number of patients		86	196	
Age	Median (SD)	70 (11.2)	67 (9.3)	0.112*
Male sex	(%)	58 (67.4)	115 (58.7)	0.185^¶^
BMI	Average	22.4	22.6	0.966*
Diagnosis				
PDAC	(%)	33 (38.4)	108 (55.1)	0.184^¶^
Bile duct ca.		20 (23.3)	31 (15.8)	
Duodenum ca.		15 (17.4)	22 (11.2)	
NET/NEC		3 (3.5)	6 (3.1)	
IPMN		10 (11.6)	17 (8.7)	
Others		5	12	
Pre-Alb	Median	3.9	3.9	0.385*
ASA-PS				
1	(%)	3 (3.5)	10 (5.1)	0.033^¶^
2		57 (66.2)	97 (49.4)	
3		26 (30.2)	89 (45.4)	
Diabetes Mellitus	(%)	34 (49.2)	67 (34.1)	0.42^¶^
BD stenting	(%)	34 (39.5)	81 (41.3)	0.794^¶^
Operating time	Average	503 min	458 min	< 0.001*
Bleeding	Median	610 g	633 g	0.234*
Required PV reconstruction		10	35	0.219^¶^
Hard pancreas	(%)	29 (33.7)	77 (39.3)	0.424^¶^
Soft pancreas	(%)	57 (66.3)	119 (60.7)	
MPD diameter	Average	4.4 mm	4.7 mm	0.334*
D-AMY				
POD1	Median (IQR)	2,416 (378–9,827)	2,800 (165–7,885)	0.583*
POD3	Median (IQR)	258 (48–1187)	305 (49–1,360)	0.487*
POPF Grade B + C	(%)	20 (23.2)	45 (23.0)	0.546^¶^
POPF Grade B	(%)	19 (22.0)	41 (21.0)	0.824
POPF Grade C	(%)	1 (1.2)	4 (2.0)	
POHS	Median	17 days	18 days	0.545*
Mortality	(%)	0	0	
Clavian–Dindo > 3a	(%)	25 (29.1)	56 (28.6)	1*

*BMI* Body Mass Index, *PDAC* pancreatic adenocarcinoma, *NET/NEC* nuroendocrine tumor/carcinoma, *ca.* carcinoma, *IPMN* intraductal papillary mucinous neoplasm, *Pre-Alb* pre-operaive albumin, *ASA-PS* American Society of Anesthetists Physical Status, *BD stenting* pre-operative bile duct stenting, *PV* portal vein, *MPD* main pancreatic duct, *D-AMY* amylase value of drainage fluid, *IAD-AMY* amylase value of inter anastomosis drainage fluid POD, post-operative day, *POPF* post-operative pancreatic fisitula, *POHS* post-operative hospital stay, *IQR* interquartile range

*Performed by Mann–Whitney U test

^¶^Performed by Chi-squrared test

**Table 2:** The summary of 176 patients with soft pancreas in B + IAD and B group

		Soft pancreas limited		*p* value
		B + IAD	B	
Number of patients		57	119	
Age	Median (SD)	70 (12.5)	70 (10.0)	0.35*
Male sex	(%)	39 (68.4)	70 (58.8)	0.22^¶^
BMI	Average	22.8	23.1	0.95*
Dx				
PDAC	(%)	13 (22.8)	36 (30.2)	0.86^¶^
Bile duct ca.		18 (31.6)	29 (24.4)	
Duodenum ca.		11 (19.3)	22 (18.5)	
NET/NEC		2 (3.5)	5 (4.2)	
IPMN		9 (15.8)	16 (13.4)	
Others		4	11	
Pre-Alb	Median	3.9	3.9	0.28*
ASA-PS				
1	(%)	3 (5.3)	10 (8.4)	0.07^¶^
2		38 (66.7)	57 (47.9)	
3		16 (28.1)	52 (43.7)	
Diabetes mellitus	(%)	19 (33.3)	32 (26.9)	0.38^¶^
BD stenting	(%)	23 (40.3)	43 (36.1)	0.59^¶^
MPD diameter	Average (IQR)	3.9 (2–5)	3.7 (2–5)	0.58*
Operation time	Average	507 min	458 min	0.001*
Bleeding	Median	708 g	831 g	0.17*
Required PV reconstruction		1	7	0.22^¶^
D-AMY				
POD1 (IU/I)	Median (IQR)	6152 (2301–13,409)	6078 (3099–15,321)	0.7*
POD3 (IU/I)	Median (IQR)	2153 (167–1530)	2777 (275–2130)	0.16*
POPF Grade B + C	(%)	19 (33.3)	42 (35.3)	0.67^¶^
POPF Grade B	(%)	16 (28.1)	38 (31.9)	0.85^¶^
POPF Grade C	(%)	3 (5.3)	4 (3.4)	
POHS	Median	22.5 days	21 days	0.81*
Clavian–Dindo > 3a	(%)	24 (42.1)	51 (42.9)	0.93^¶^

*BMI* Body Mass Index, *PDAC* pancreatic adenocarcinoma, *NET/NEC* nuroendocrine tumor/carcinoma, *ca.* carcinoma, *IPMN* intraductal papillary mucinous neoplasm, *Pre-Alb* pre-operaive albumin, *ASA-PS* American Society of Anesthetists Physical Status, *DM* diabetes mellitus, *BD stenting* pre-operative bile duct stenting, *PV* portal vein, *MPD* main pancreatic duct, *D-AMY* amylase value of drainage fluid, *IAD-AMY* amylase value of inter anastomosis drainage fluid, *POD* post-operative day, *POPF* post-operative pancreatic fisitula, *POHS* post-operative hospital stay, *IQR* interquartile range

*Performed by Mann–Whitney U test

^¶^Performed by Chi-squrared test

### The incidence of pancreatic fistula

Among the 282 patients with hard and soft pancreatic textures, PF grade B and C occurred in 19 (22%) and 1 (1.2%) patient(s), respectively, in group B + IAD and in 41 (21.0%) and 4 (2.0%) patients, respectively, in group B. Clinically relevant POPF was observed in 20 (23.2%) and 45 (23.0%) patients in the B + IAD and B groups, respectively (*p* = 0.546). Surgical complications diagnosed as greater than Clavien–Dindo 3a occurred in 25 (29.1%) and 56 (28.6%) patients in the B + IAD and B groups, respectively (*p* > 0.99). No postoperative mortality was observed in any group. The median postoperative hospital stay (POHS) was 17 and 18 days in the B + IAD and B groups, respectively (*p* = 0.545) (Table 1).

Among the 176 patients with a soft pancreas, 57 underwent B + IAD and 119 patients underwent B-only. The data are shown in Table 2. PF grades B and C occurred in 16 (28.1%) and 3 (5.3%) patients, respectively, in the B + IAD group and in 38 (31.9%) and 4 (3.4%) patients, respectively in the B group (*p* = 0.67). Surgical complications diagnosed as greater than Clavien-Dindo 3a occurred in 24 (42.1%) and 51 (42.9%) patients in the B + IAD and B groups, respectively (*p* = 0.925). The median POHS was 22.5 and 21 days in the B + IAD and B groups, respectively (*p* = 0.81).

### The effect of the inter-anastomosis drainage tube

**Fig. 2:**
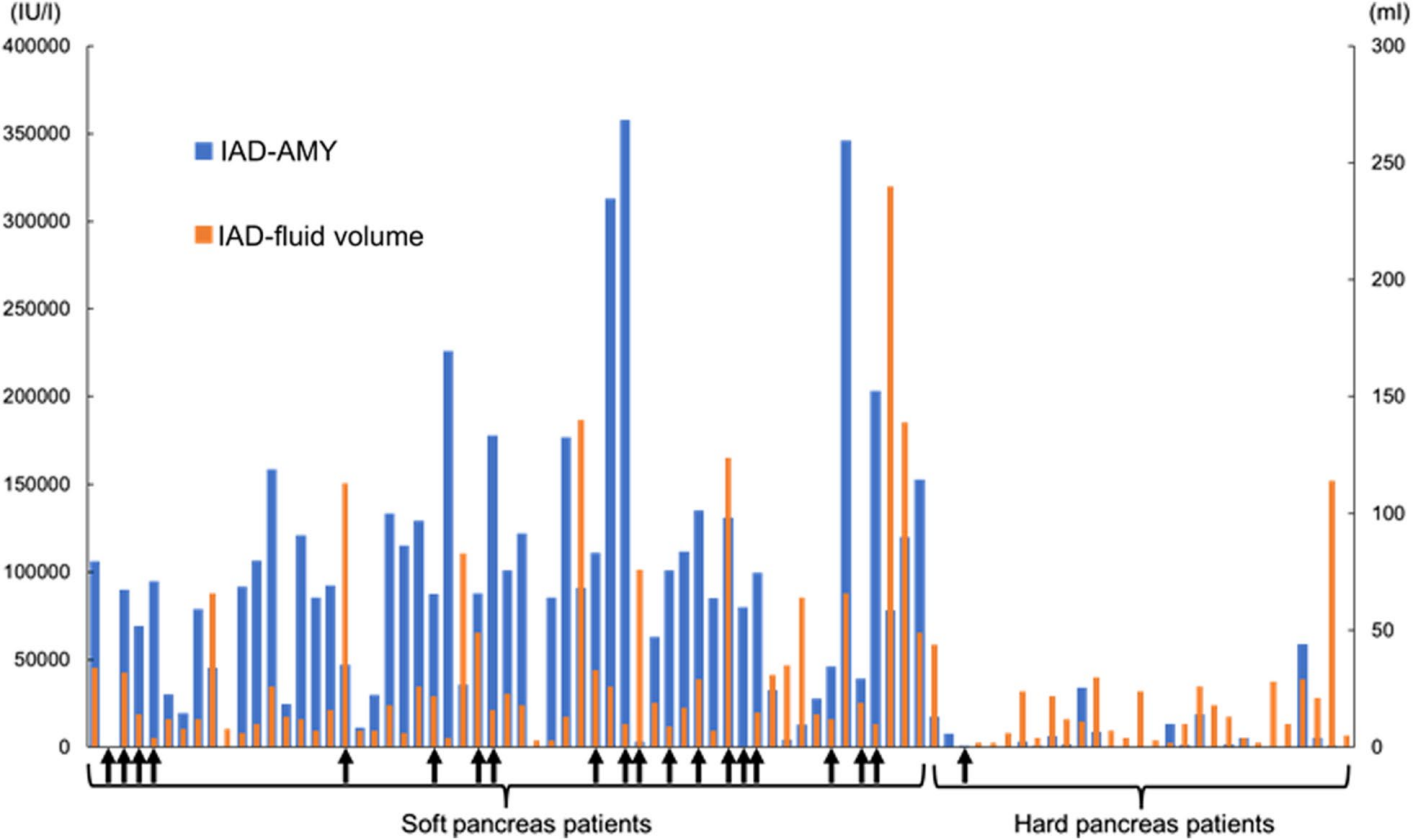
Amylase value and the drainage volume of each patient in the IAD + B group. Bar-graph (left side) indicates the amylase value of IAD tube collection, and the right side indicates the volume of the IAD collection. Arrows revealed the existence of grade B or C pancreatic fistula

The amount of IAD drainage fluid and amylase level for both the soft and hard pancreas is shown in Fig. 2. In 86 patients (B + IAD group), the median maximum volume of drainage fluid was 14 mL/day (range 0–240 mL). The daily maximum drainage was significantly higher in patients with a soft pancreas than in those with a hard pancreas (16.5 mL/day vs. 10.0 mL/day; *p* = 0.012). The amylase level in the IAD drainage fluid was significantly higher in patients with a soft pancreas than in those with a hard pancreas (median 90,900 IU/L vs. 1,634 IU/L; *p* < 0.0001). However, the amylase level of external peritoneal drainage did not change in the B + IAD group compared to the B group, both in total cohort (Table1) and soft pancreas cohort (Table 2). All patient data sets used in this study are available in Additional File 1.

## Discussion

This study examined the effect of our novel concept of indwelling an IAD suction tube to prevent POPF during PD reconstruction. This method is expected to reduce undesired pancreatic juice from the branched pancreatic duct and duct-to-mucosa anastomosis and compensate for the shortcomings of Blumgart-type pancreatojejunostomy. This method successfully collected notably amylase-rich fluids in the inter-anastomosis space. However, we could not identify meaningful improvements in terms of the incidence of clinically relevant PF and POHS duration.

Our IAD tube method showed, for the first time, the existence of a substantial volume (maximum of 14.0 mL/day [median] in total 86 patients from the B + IAD group and 16.5 mL/day in patients with a soft pancreas) of undesirable fluid in the interspace between the jejunal wall and pancreatic parenchyma that contained markedly high amylase levels (median value: 90,900 IU/L in patients with a soft pancreas). This undesired fluid could be considered the origin of PF. However, it is difficult to ascertain whether fluid effluxed from the branch duct was a minor leakage from the duct-to-mucosa anastomosis. From this result, we suggest that the soft pancreas produced a high amount of amylase-rich pancreatic juice and the operator’s subjective palpation during the procedure regarding whether the pancreas was soft or hard seems correct.

**Fig. 3:**
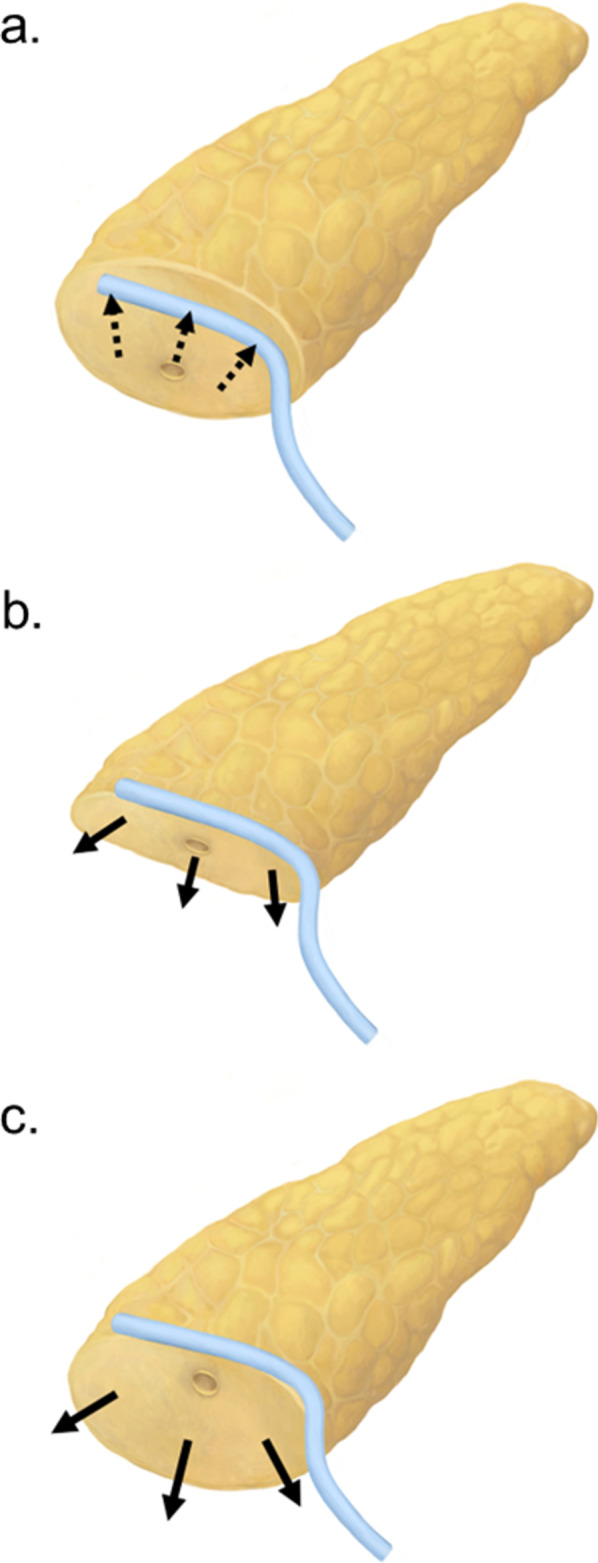
MPD location types and IAD tube placement in each cases. The inter-anastomosis drainage (IAD) in Blumgart-type pancreatojejunostomy works effectively when the pancreas resection surface is wide (not small) and the main pancreatic duct locates on the dorsal side because IAD tube placed fine in water-tight manner (**a**). IAD tube placement did not work well when the resection surface is thin or small (**b**) and the main pancreatic duct locate on the ventral side (**c**)

Although the IAD tube successfully collected fluid with notably high amylase levels at the inter-anastomosis space in Blumgart-type pancreatojejunostomy, we could not identify any meaningful improvements in clinically relevant PF. PF grades B and C occurred in 28.1% and 5.3% of patients, respectively, in the B + IAD group and in 31.9% and 3.4% of patients, respectively, in the B group. Our initial report on 17 patients using this novel IAD tube method revealed a significant reduction in the incidence rate of PF grade B or C (the incidence rate was 5.9%; 1 patient out of 17 patients) [15]. The POHS did not reduce in the B + IAD group compared to the B group, both in POPF developed and non-developed cases. The POHS in patients who experienced POPF was 29 days and 35 days in the non-IAD (B group) and the B + IAD groups, respectively (*p* = 0.087). The IAD tube functions effectively when the pancreatic cut margin is wide and the MPD is located on the dorsal side (Fig. 3a). Conversely, when the surface area of the pancreatic stump is small or the MPD is located ventrally (Fig. 3b, c), the insertion of IAD tube is troublesome and the result is undesirable.

The reason why the IAD tube method did not improve after the first report may be due to three reasons. The first reason is the technical difficulty in the placement of the IAD tube at the inter-anastomosis space between the jejunal wall and the pancreatic parenchyma, especially in cases including a thin pancreas stump. For the IAD tube to exert a pronounced effect in order to protect leakage from the branch duct, the tube needs to be placed in a water-tight-seal manner. This water-tight seal was made using 2 or 3 Blumgart suture knots at the jejunal surface, while embedding the IAD tube above the duct-to-mucosa anastomosis [15] (Fig. 1b, c). If the IAD tube had been able to collect pancreatic leakage, we could expect a decrease in amylase level of the abdominal drain, but this was not confirmed. This may be due to the insufficient water-tight seal. Moreover, this is occasionally a highly challenging problem especially in the case of a small pancreatic cross-section. When the above side of pancreatic stump is thin and same as the width of IAD tube, the tube placement with a water-tight seal is theoretically impossible. In fact, the 17 patients reported in our first report [15] were operated by staff surgeons engaged in pancreatic surgery. This suture technique, creating a water-seal adhesion, is technically difficult for trainees. In fact, the operating time was longer in the B + IAD group than the B group in the soft pancreas cohort. This may reflect the reconstruction required more times to prudent suture techniques.

Second, the location of the MPD in the stump was important for effective collection. When the MPD was located on the ventral side of the stump, it was quite difficult to place the IAD tube above the duct-to-mucosa anastomosis similar to that in patients with thin parenchyma. Moreover, the fluid that leaks below the anastomosis might not be collected effectively by the IAD tube because the IAD tube was only placed above. This might also indicate that the IAD tube could not entirely collect the undesired fluid discharged from both the pancreatic stump and the incomplete duct-to-mucosa anastomosis. When the MPD is located ventrally, the IAD tube can be placed below the duct-to-mucosa anastomosis. In such cases, the space under the anastomosis is important, and if the space is narrow, it becomes difficult to maintain the water-tight seal by Blumgart anastomosis. The situation is similar even when the MPD is located dorsally.

The third reason was that the IAD tube placement may prevent tissue adhesion, which is important for the cure of PF. Though IAD successfully collected the amylase-rich fluids, foreign substances may delay self-healing. In fact, the POHS in patient who developed POPF was longer in the B + IAD group than in the B group (without IAD). This may be due to the prolonged wound healing caused by the IAD tube placement, which we consider a drawback of IAD. The median maximum amount of IAD tube collection was 14.0 mL/day and more than 100 mL/day was recorded in some patients. This observation may suggest that the collections from the IAD tube included not only pancreatic juice but also enteric fluids that contained abundant enterobacteria. Though IAD tube placement may trigger bacterial infection around the pancreatojejunostomy, we did not experience any fatal infectious complications during the study period.

To overcome the technical difficulties associated with IAD tube placement, a simpler and more reasonable method is required to allow the pancreatic juice to drain into the jejunum. Recent studies have suggested that the healing of PF requires the amylase-rich fluid to drain spontaneously into the jejunal lumen [12]. To achieve spontaneous drainage at an early stage after PD, the intestinal internal pressure needs to be kept low by the placement of an enteric drainage tube near the pancreatojejunostomy. High intestinal internal pressure may induce the outflow of enteric juice and bacterial infection, which activates proteases. Subsequently, the intentional drainage route in the jejunal wall may enable the fluid to spontaneously drain into the jejunum from the early postoperative period without the placement of an IAD tube. To accomplish this, two key factors, tight adhesion between the jejunal wall and pancreatic stump and low pressure inside the jejunum, are required.

In the present study, the incidence of clinically relevant PF (grade B or C) was 23% in the total cohort and 34% in the soft pancreas cohort. These values were relatively high compared to other studies on Blumgart-type anastomosis [6, 9]. However, POHS did not change significantly compared to these studies [6, 8, 16]. In fact, we achieved 17 days of POHS after PD without mortality. According to the ISGPF grading system [13], PF grade B requires a change in the postoperative management; thus, drains were either left in place for more than 3 weeks or replaced by endoscopic or percutaneous procedures. We generally performed an early assessment of the obstruction of drainage tubes on POD 3 or 4 and replacement of tubes under radioscopy if the drainage was not effective. Patients who underwent drain tube replacement or for whom the drainage site was changed under radioscopy were judged to have PF grade B. These facts may be responsible for the increased number of patients diagnosed with PF grade B in our study. Even if a patient experiences clinically relevant PF, the duration of POHS is important.

This study has some limitations. First, this is a retrospective study from a single institute, i.e., a teaching hospital. The sample size was limited, and each procedure was performed by several surgeons, including trainees. All procedures were guided by three staff surgeons; however, maintaining reproducibility was not easy due to the technical difficulty of IAD tube insertion. Second, the judgement of soft or hard pancreatic texture was made subjectively by the surgeons. Potential selection bias is difficult to eliminate, and a precise method to assess pancreas status is warranted. Third, since it is difficult to ascertain whether the cause of PF is due to an unsuccessful anastomosis or leakage from the branched pancreatic duct, it is impossible to know the details of how the IAD worked. Forth, pancreatic exocrine secretion depends on the size of remnant pancreas, acinar cell function, and number of detailed branch ducts in the stump. Patients with low exocrine potential may not require this IAD method. Therefore, further studies are necessary to clarify the relationship between remnant pancreatic function and the effect of IAD tube insertion.

## Conclusions

This series highlights the nature of the space between the jejunal wall and pancreatic parenchyma in Blumgart-type pancreatojejunostomy using IAD tube drainage. Markedly high amylase-rich fluids were observed in patients with a soft pancreas using IAD suction tube drainage, and these collections may be essential for the development of PF. However, IAD tube method utilized with Blumgart anastomosis did not improve the incidence of clinically relevant PF. For a safer PD with Blumgart pancreatojejunostomy, additional improvements are required to overcome the pancreatic juice leakage in the inter-anastomosis space.

## Supplementary Information

**Additional file 1.** Pre-operative factors. B+IAD group.

## Data Availability

All data generated or analysed during this study are included in its Additional files.

## Abbreviations

IAD: Inter-anastomosis drainage
PF: Pancreatic fistula
PD: Pancreatoduodenectomy
POPF: Post-operative pancreatic fistula
MPD: Main pancreatic duct
B: Blumgart-type anastomosis
ISGPF: International Study Group on Pancreatic Fistula
POHS: Post-operative hospital stay
